# Visualization of odor-induced neuronal activity by immediate early gene expression

**DOI:** 10.1186/1471-2202-13-140

**Published:** 2012-11-05

**Authors:** Asim K Bepari, Keisuke Watanabe, Masahiro Yamaguchi, Nobuaki Tamamaki, Takebayashi Hirohide

**Affiliations:** 1 Department of Morphological Neural Science, Graduate School of Medical Sciences, Kumamoto University, Kumamoto, Japan; 2 Division of Neurobiology and Anatomy, Graduate School of Medical and Dental Sciences, Niigata University, Niigata, Japan; 3 Department of Physiology, Graduate School of Medicine, University of Tokyo, Tokyo, Japan; 4 PRESTO, Japan Science and Technology Agency (JST), Kawaguchi, Saitama, Japan

**Keywords:** Olfaction, Odorant map, Olfactory bulb, C*nga2*, Immediate early gene (IEG)

## Abstract

**Background:**

Sensitive detection of sensory-evoked neuronal activation is a key to mechanistic understanding of brain functions. Since immediate early genes (IEGs) are readily induced in the brain by environmental changes, tracing IEG expression provides a convenient tool to identify brain activity. In this study we used in situ hybridization to detect odor-evoked induction of ten IEGs in the mouse olfactory system. We then analyzed IEG induction in the cyclic nucleotide-gated channel subunit A2 (*Cnga2*)-null mice to visualize residual neuronal activity following odorant exposure since CNGA2 is a key component of the olfactory signal transduction pathway in the main olfactory system.

**Results:**

We observed rapid induction of as many as ten IEGs in the mouse olfactory bulb (OB) after olfactory stimulation by a non-biological odorant amyl acetate. A robust increase in expression of several IEGs like *c-fos* and *Egr1* was evident in the glomerular layer, the mitral/tufted cell layer and the granule cell layer. Additionally, the neuronal IEG *Npas4* showed steep induction from a very low basal expression level predominantly in the granule cell layer. In *Cnga2*-null mice, which are usually anosmic and sexually unresponsive, glomerular activation was insignificant in response to either ambient odorants or female stimuli. However, a subtle induction of *c-fos* took place in the OB of a few *Cnga2*-mutants which exhibited sexual arousal. Interestingly, very strong glomerular activation was observed in the OB of *Cnga2*-null male mice after stimulation with either the neutral odor amyl acetate or the predator odor 2, 3, 5-trimethyl-3-thiazoline (TMT).

**Conclusions:**

This study shows for the first time that in vivo olfactory stimulation can robustly induce the neuronal IEG *Npas4* in the mouse OB and confirms the odor-evoked induction of a number of IEGs. As shown in previous studies, our results indicate that a CNGA2-independent signaling pathway(s) may activate the olfactory circuit in *Cnga2*-null mice and that neuronal activation which correlates to behavioral difference in individual mice is detectable by in situ hybridization of IEGs. Thus, the in situ hybridization probe set we established for IEG tracing can be very useful to visualize neuronal activity at the cellular level.

## Background

Chemosensory cues from the environment are detected by the peripheral nervous system which then transmits sensory information to the central nervous system and activates discrete neuronal ensembles. Studies on patterns of neuronal activation provide useful insights into roles of different neuronal population and brain regions in regulating distinct animal behaviors. IEG expression is induced rapidly when neurons are activated by membrane depolarization, seizure or some sensory signals [1] and the expression pattern of IEGs is a convenient tool for visualization of neuronal activities [2-4]. Neuronal IEGs comprise of several categories including transcription factors (*c-fos, Fosb, c-jun, Junb, Egr1, Egr2, Egr3, Npas4, Nr4a1, Nr4a2*, etc.) and postsynaptic proteins (*Arc, Homer1a*, etc.). Previous studies which used long-term potentiation (LTP) or long-term enhancement paradigm indicated that different IEGs have different thresholds for transcriptional induction; *c-fos,* for instance, has a high threshold compared to that of *Egr1*[5,6]. Guzowski *et al.*, (2001) found a higher responsiveness of *Arc* than that of *c-fos* and *Egr1* in the rat hippocampus after spatial learning [7].

Olfactory sensory neurons (OSNs) in the main olfactory epithelium (MOE) can detect a vast array of odorous molecules by the olfactory receptors (ORs) [8]. OSNs make synapses directly to second-order neurons in the central nervous system. Each OSN projects to a single glomerulus and the OSNs which express a particular OR usually converge to a single glomerulus both in the medial and lateral halves of the OB and thus OB glomeruli form a topographical map of ORs [9,10]. Consequently, afferent inputs through OSNs trigger activity in the OB which is often traced by specific induction of IEGs. However, it should be noted that in addition to peripheral stimulation, centrifugal inputs can significantly influence the pattern of activity in the OB, particularly in the granule cell layer [11-14].

Interaction of odorants with ORs in vertebrate OSNs activates the olfaction-specific G protein (G_olf_) which in turn stimulates other components of the signaling cascades including the adenylyl cyclase type III (ACIII) and the olfactory cyclic nucleotide-gated channel (CNGC) [15]. Previous knockout mice studies have confirmed that the cAMP signaling pathway plays the key role for detection of odorants [16,17]. Belluscio *et al.* (1998) reported that most G_olf_-deficient mice showed neonatal mortality [17]. In addition, electro-olfactogram (EOG) recordings, which measure electrical activity detected by an electrode placed on the olfactory epithelium, indicated severe reduction in odor-evoked response in G_olf_-deficient mice [17]. The odorant-induced EOG response was found to be completely ablated and the odorant-dependent avoidance learning was impaired in ACIII mutant mice [18]. The mice which have mutation in the cyclic nucleotide-gated channel subunit A2 (*Cnga2*) gene also show general anosmia [19]. In 1 day-old *Cnga2*-mutants there was no detectable EOG response even when the olfactory epithelium was exposed to complex olfactory stimuli such as mouse urine (conspecific odor cues) or coyote urine (predator odor cues) [19]. Behavioral studies in adult *Cnga2*-null male mice showed that they fail to mate or fight and it is suggested that the MOE has an essential role in regulating these social behaviors [20]. Although the mutant male mice failed to show preference for female urine [20], it remains unclear whether the female urine odor activated the OB glomeruli or not. Intriguingly, Lin *et al.* (2004) showed that several odorants, including putative pheromones, were behaviorally detected by the *Cnga2-*null mice and electrophysiological and immunohistochemical studies revealed that those odors indeed produced responses in the MOE, OB and piriform cortex (PC) in the mutants [21].

For mapping neuronal activity using IEGs two important criteria should be the low basal expression and the high induction of the IEG being used. It is also advantageous to analyze several IEGs since the induction thresholds of IEGs vary depending on the IEG, the stimulus and the tissue. In this present study we used ISH to analyze expression patterns of ten IEGs in the mouse brain using different odor stimuli and compared inducibility and sensitivity of these IEGs for detection of sensory-evoked neuronal activities. We then asked how disruption of the cAMP signaling cascade in the olfactory pathway affects neuronal activation using *Cnga2*-null male mice which show general anosmia and sexual deficits. We exposed the mutants to different odorants including a predator odor and female odors to observe behavioral responses and the pattern of brain activities mediated by any CNGA2-independent olfactory signaling pathway(s) using ISH of IEGs.

## Results

### Odor-evoked rapid induction of ten activity-dependent genes in the mouse OB

**Figure 1:**
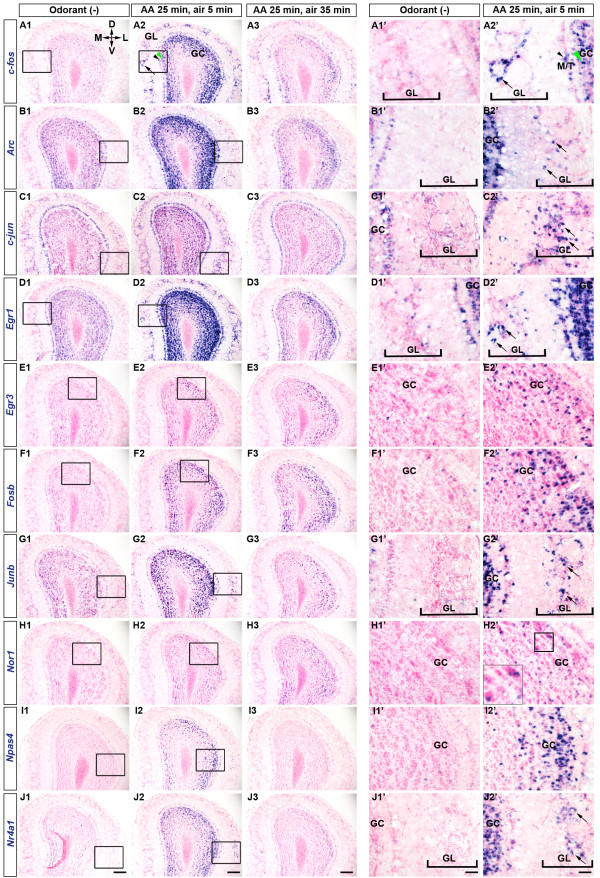
**Odorant (amyl acetate) exposure induced the expression of IEGs in the mouse OB.** Mice were exposed to overhead airflow for 2 h and then to the test odorant (amyl acetate) for 25 min (5-min exposures with 5-min intervals). The ISH of coronal sections of OB indicated low expression levels of ten IEGs in mice immediately after the 2-h air exposure, (Odorant (−), **A1-J1**, **A1’-J1’**). All these ten IEGs were induced in the mouse OB after 30 min of odor onset (AA 25 min, air 5 min, **A2-J2**, **A2’-J2’**). Boxed areas in **A1-J1** and **A2-J2** are magnified in **A1’-J1’** and **A2’-J2’**, respectively. Inset in **H2’** is a magnified view of the boxed area. Odor-evoked induction of IEG expression was transient and expression levels of most of the IEGs declined within 60 min of initial odorant exposure (AA 25 min, air 35 min, **A3-J3**). Arrows indicate GL, black arrowheads indicate M/T and green arrowheads indicate GC. AA, amyl acetate; GL, Glomerular layer; M/T, Mitral/Tufted cell layer; GC, Granule cell layer. D, Dorsal; V, Ventral; M, Medial; L, Lateral. Scale bars: (**A1-J3**) 200 μm, (**A1’-J2’**) 50 μm.

**Figure 2:**
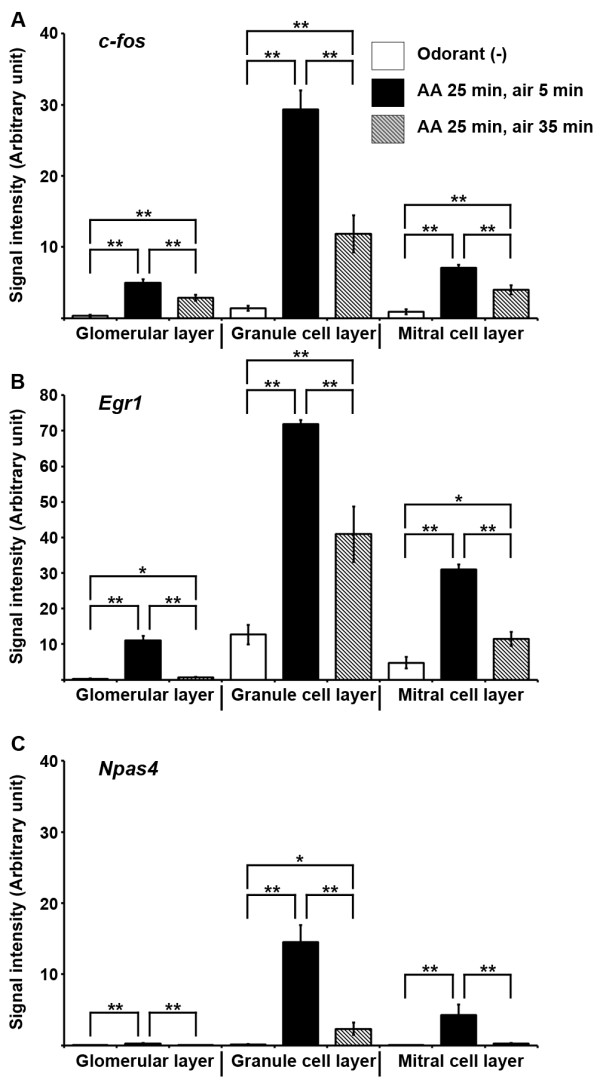
**Quantification of odor-evoked IEG induction in the mouse OB.** Signal intensity (arbitrary unit) of *c-fos* (**A**), *Egr1* (**B**) and *Npas4* (**C**) was calculated as the percentage of area positive for ISH signals in respective layers of the OB. Columns represented mean ± SEM. Seven to eight bulbs (approximately from + 4.5 mm bregma to + 4 mm bregma) from two to three mice were analyzed. Student's *t*-test was performed to compare means. ** Difference between groups was highly significant (p ≤ 0.01). * Difference between groups was significant (p ≤ 0.05).

To visualize neuronal activities in response to environmental changes we used ISH of activity-dependent genes in the mouse brain following presentation of odor stimuli. To minimize the level of ambient odorants, test animals were kept under overhead air flow for 2 h before the odorant exposure. The mice which were sacrificed immediately after the 2-h air exposure were treated as controls, the ‘Odorant (−)’ group. IEG expression levels were found to be very low in control mice (Figure 1A1-J1, A1’-J1’, Figure 2). For olfactory stimulation we first used amyl acetate since it is a standard nonbiological odorant which produces strong and repeatable responses [22,23]. As many as ten IEGs were induced when the mice were exposed to amyl acetate for 25 min (5-min exposures with 5-min intervals) and sacrificed after 30 min from the odor onset (AA 25 min, air 5 min) (Figure 1A2-J2, A2’-J2’, Figure 2). Expression levels of most of these IEGs decreased substantially within 60 min of the odor onset (AA 25 min, air 35 min) (Figure 1A3-J3, Figure 2), indicating that the odor-evoked IEG induction was transient. Nevertheless, odorant-induced higher expression levels of *Egr3* (Figure 1E1-E3), *Fosb* (Figure 1F1-F3) and *Nor1* (Figure 1H1-H3) seemed to be sustained at least for 60 min from the initial odor presentation.

The main projection neurons in the mouse OB are the mitral/tufted cells and there are several types of interneurons such as granule cells and periglomerular cells. We could visualize activation of spatially segregated glomeruli by the strong *c-fos* expression in periglomerular cells (arrow, Figure 1A2, A2’) even though the ISH signals spanned the entire glomerular layer. The *c-fos* mRNA signals were abundant in the mitral/tufted cell layer (black arrowhead, Figure 1A2, A2’) and very dense signals were observed in the superficial aspects of the granule cell layer (green arrowhead, Figure 1A2, A2’). Similarly, we observed robust induction of *Arc*, *c-jun*, *Egr1* and *Junb* after the odorant exposure (Figure 1). It appeared that at the activated glomeruli the *Egr1* induction took place in the majority of periglomerular cells (arrows, Figure 1D2’), whereas, *Arc*, *c-jun* and *Junb* were upregulated in subsets of periglomerular cells (arrows, Figure 1B2’, C2’, G2’, respectively).

In control mice signals of *Egr3*, *Fosb*, *Nor1*, *Npas4* and *Nr4a1* mRNAs were barely detectable either in the glomerular layer or in the mitral/tufted cell layer although a small fraction of granule cells were positive for these IEGs (Figure 1E1,E1’, F1,F1’, H1,H1’, I1,I1’, J1,J1’). After the amyl acetate exposure, significant induction of these five IEGs was apparent in the granule cell layer (Figure 1E2,E2’, F2,F2’, H2,H2’, I1,I1’, J2,J2’) and sparse signals appeared in a few periglomerular cells and the mitral/tufted cells (Figure 1J2’, data not shown).

In our subsequent experiments we analyzed induction patterns of *c-fos*, the most widely used IEG, along with *Npas4* since the activity-dependent induction of *Npas4* has not been previously reported in the mouse olfactory system. It was interesting to note that after 30 min of odor onset, *Npas4* expression was robustly increased from a very low basal level and then, there was a steep decline within 60 min of odor onset (Figure 1I1-I3, Figure 2C). Our results indicated that expression patterns of IEGs in the mouse OB varied considerably at the basal condition and a single session of odorant exposure was sufficient to induce expression of the ten IEGs we examined.

### Different odorants produce differential responses in the mouse brain

**Figure 3:**
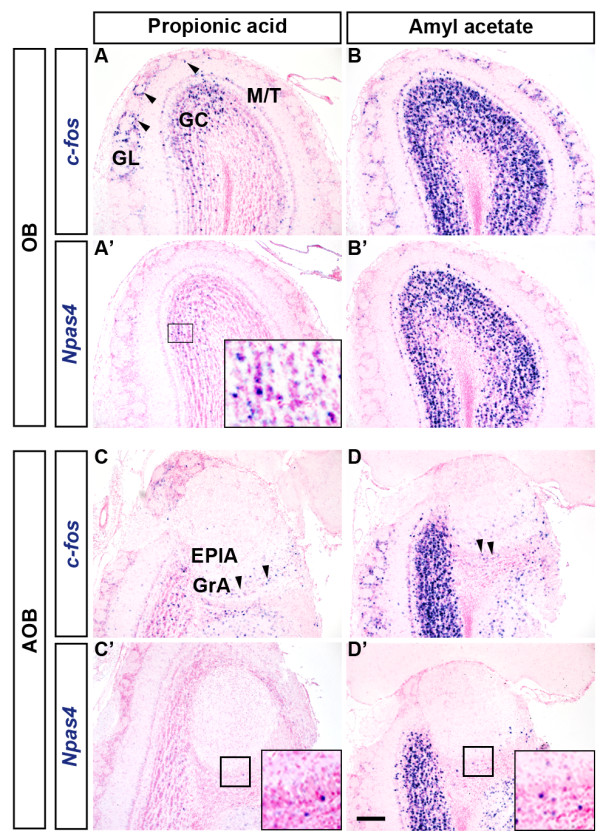
**Comparison of IEG induction patterns in response to two different odorants.** Mice were sacrificed after the 30-min continuous exposure to the test odorant. (**A, A’**) Propionic acid activated several glomeruli specifically in the dorsal OB (arrowheads in A). Induced expression of *Npas4* was observed only in the granule cell layer (A’, inset). (**B, B’**) A large number of glomeruli were activated by amyl acetate. *Npas4* induction was apparent only in the granule cell layer (B’). (**C-D’**) Patterns of IEG induction in the AOB after odorant exposure. Arrowheads indicate *c-fos* induction in the granule cell layer of the AOB (C, D). Only a slight induction of *Npas4* was observed in the AOB (C’, D’, insets). GL, Glomerular layer; M/T,Mitral/Tufted cell layer; GC, Granule cell layer; GrA, Granule cell layer of the AOB; EPlA, External plexiform layer of the AOB. Scale bar: 200 μm.

The OB glomeruli are spatially organized into the dorsal (D_I_ and D_II_) and the ventral (V) domains and different odorants activate distinct sets of glomeruli in mice [24]. Therefore, we used two different odorants for olfactory stimulation and then observed the neuronal activation pattern by the ISH of activity-dependent genes. When we exposed mice to propionic acid, an aliphatic acid with pungent odor, we found that only a small number of glomeruli were strongly activated at the dorsomedial aspect of the anterior OB (arrowheads, Figure 3A, A’) [25,26]. There were strong signals of *c-fos* mRNAs in periglomerular cells around the glomeruli which were presumed to be specifically activated by propionic acid. In addition, the induced expression of *c-fos* was observed in the mitral/tufted cell layer and the granule cell layer below the activated glomeruli (Figure 3A). Using optical imaging a previous study also showed that propionic acid specifically activated the anteromedial domain of the mouse OB [27]. On the other hand, amyl acetate, a strong neutral odorant, activates many glomeruli both in the dorsal and the ventral OB [25,28-30]. We also found that amyl acetate robustly induced *c-fos* expression in a large number of periglomerular cells, mitral/tufted cells and granule cells in both the dorsal and ventral aspects of the OB (Figure 3B). Induction of *Npas4* was evident mainly in the granule cell layer of the OB for both propionic acid and amyl acetate (Figure 3A’, B’). Accessory olfactory bulb (AOB) neurons were found to respond to the volatile, conspecific as well as allospecific odor cues [31,32]. Our results were in agreement with the emerging evidence for the overlapping functions of the mouse OB and the AOB in processing olfactory cues [33,34]. Using ISH of IEGs we found that odorants like amyl acetate and propionic acid, which are not pheromones, induced *c-fos* expression not only in the OB but also in the AOB (Figure 3A, B, C, D). Induced expressions of *Npas4* were evident in the OB (Figure 3A’, B’) for both of these odorants although *Npas4* was only slightly induced in the AOB (Figure 3C’, D’, insets). Therefore, these data indicate that the IEG induction patterns we observed were odorant-specific and by tracing IEG expression using ISH, it is possible to demarcate brain activities with very high spatial resolution.

### IEG induction demarcates the flow of olfactory information in the higher order brain regions

**Figure 4:**
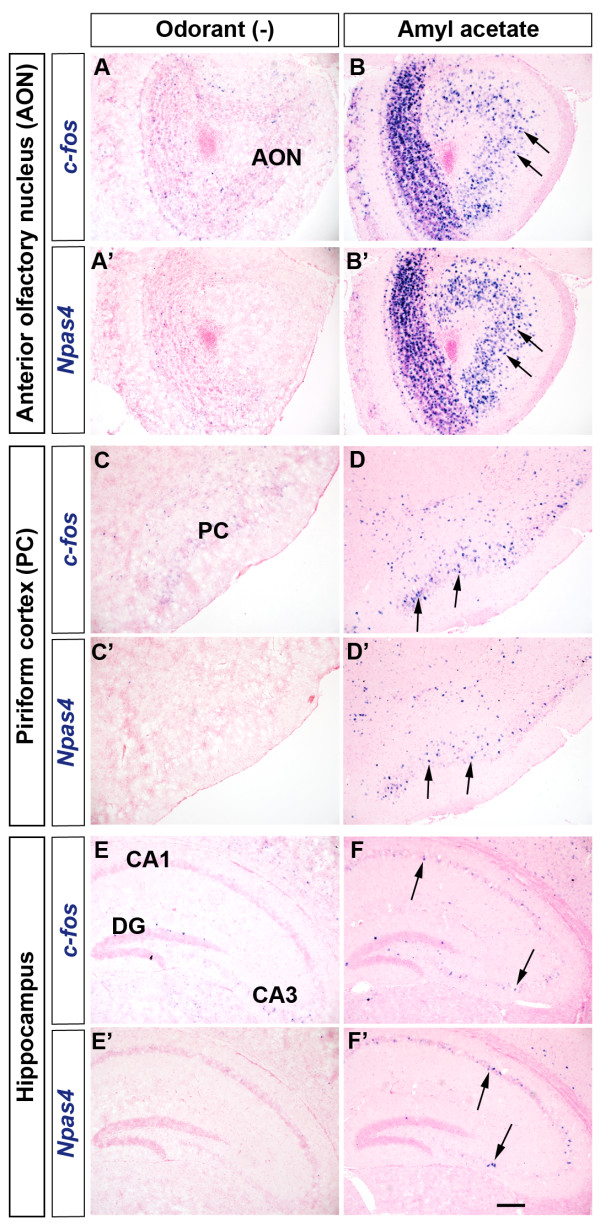
**Odorant exposure induced activity-dependent gene expression in different brain regions.** Odorant exposure induced expression of *c-fos* (**A-F**) and *Npas4* (**A’-F’**) in the AON (arrows, **A-B’**), the PC (arrows, **C-D’**) and the hippocampus (arrows, **E-F'**). DG, Dentate gyrus. Scale bar: 200 μm.

Olfactory information is conveyed to and processed in a number of cortical and subcortical brain regions including the anterior olfactory nucleus (AON), the PC, the amygdala and the entorhinal cortex [8,35]. We found that the increase in the expression of these IEGs in AON (arrows, Figure 4A-B’) paralleled to the activation of OB neurons. It is known that in the PC pyramidal neurons receive direct input from mitral/tufted cells of the OB. Consequently, odorant exposure activates unique but overlapping subsets of neurons in the PC [36]. As expected, we observed odorant-induced increase in expression of IEGs in the layer 2/3 of the PC where cell bodies of pyramidal neurons are located (arrows, Figure 4D, D’).

Owing to the intimate connection between olfaction and memory, the hippocampus has been of great interest for studying olfactory memory. We found that the exploration of odor cues only for a brief period significantly induced the expression of several IEGs in the mouse hippocampus (Figure 4E-F', data not shown). Our ISH data clearly indicate that odor stimulus not only triggered the robust induction of IEGs in the mouse OB but also conspicuously increased the expression of these genes in various brain regions which are involved in olfactory signal processing.

### Individual differences in sexual stimuli-induced neuronal activities in *Cnga2*-null male mice

**Figure 5:**
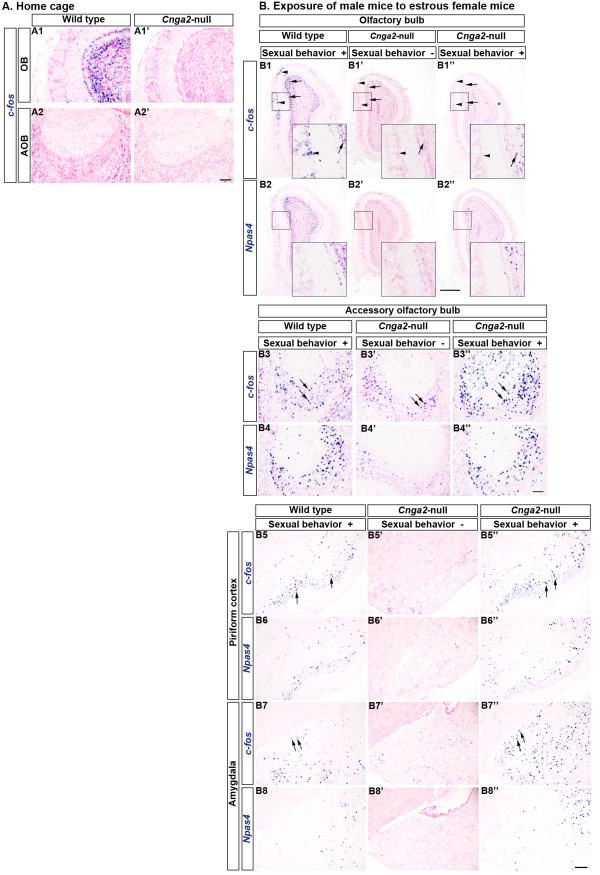
**Individual differences in induction of activity-dependent genes in *Cnga2*-null mice after exposure to female mice.A.** Expression of *c-fos* in mice which were sacrificed from their home cages without any odorant exposure. Significantly reduced expression levels of *c-fos* were observed in the OB (A1’) and AOB (A2’) of *Cnga2*-null male mice compared to that of wild type male littermates (A1, A2, respectively). **B**. Induction of *c-fos* expression in male mice which were exposed to estrous female mice. Arrowheads indicate the glomerular layer and arrows indicate the granule cell layer. Sexual stimulation by female mice induced expression of IEGs in the wild type OB (B1, B2). IEG induction was almost absent in the *Cnga2* mutants which did not show sexual behaviors (B1’, B2’). IEG induction occurred in the OB, mainly in the granule cell layer, of the *Cnga2*-null male mice which showed sniffing and mounting behaviors (B1”, B2”). Insets in (B1-B2”) show magnified views of the boxed areas. IEG induction occurred in the AOB of male mice exposed to female mice (arrows, B3-B4”). (B5-B6”) Induction of IEGs in the PC (arrows) after exposure to female mice. Both in the wild type mice (B5, B6) and the mutants (B5”, B6”) which showed sexual behaviors, expression of IEGs was induced in the PC. IEG induction did not occur in the PC of *Cnga2*-null mice (B5’, B6’) which did not show sexual behaviors. (B7-B8”) Induction of IEGs in the MePD (arrows) after exposure to female mice. Both in the wild type mice (B7, B8) and the mutants (B7”, B8”) which showed sexual behaviors, expression of IEGs was induced in the MePD. The IEG induction did not occur in the MePD of *Cnga2*-null mice (B7’, B8’) which did not show sexual behaviors. Scale bars: (A1-A2’ and B3-B4”) 100 μm, (B1-B2”) 500 μm, (B5-B8’) 200 μm.

It is interesting that most *Cnga2*-null male mice show neonatal mortality, general anosmia and deficits in sexual behaviors, however, a small number of the surviving male mutants can mate successfully [19,20]. We tested the hypothesis whether the positive sexual behavior observed in some *Cnga2-*null male mice is correlated with a concurrent activation of the main olfactory system. In the home cage, mice experience many ambient odorants which are known to produce dense *c-fos* mRNA signals in olfactory structures (Figure 5A1) [28]. First we checked the extent of IEG expression by such ambient odorants in the OB of male *Cnga2*-null mice. Figure 5A depicts clear differences between the mutant and the wild type littermates in *c-fos* induction patterns when the animals were taken from the home cage immediately before sacrifice. In the OB *c-fos* expression level was very low in mutants compared to that of wild type littermates (Figure 5A1,A1’). However, we observed strong *c-fos* signals in a few isolated glomeruli in the mutant OB (Additional file 1: Figure S1B3). Expectedly, *c-fos* mRNA signals were practically absent in the AOB (Figure 5A2,A2’) in both the wild type and the mutant mice. Baker *et al.* (1999) and Lin *et al.* (2004) previously reported dramatically reduced tyrosine hydroxylase (TH) immunoreactivity, a marker for afferent activity, in most of the typical OB glomeruli in CNGA2-deficient mice [21,37]. Nevertheless, in mutant mice strong TH staining was evident in a number of discrete glomeruli including the necklace glomeruli which are found at the posterior OB and are innervated by OSNs expressing a specific guanyl cyclase (GC-D) and a phosphodiesterase, PDE2 [21,37,38]. In *Cnga2*-null mice we observed that *Th* mRNA expression was also significantly downregulated in most of the glomeruli while strong expression was retained only in a small number of glomeruli, presumably the necklace glomeruli (Additional file 1: Figure S1B1, B2). Since CNGA2 is expressed in almost all typical glomeruli, but not in necklace glomeruli which use cGMP as a second messenger instead of cAMP for olfactory signal transduction, our results supported the view that the cAMP pathway plays the key role for activation of the majority of ORNs and that olfaction is highly attenuated in the *Cnga2*-null mice [19,21,37].

To check whether the main olfactory system in *Cnga2*-null male mice is unable to detect conspecific cues from female mice, we exposed *Cnga2*-null male mice and wild type male littermates to estrous female mice. Wild type mice started chemoinvestigation (sniffing/licking) of the female anogenital regions almost instantly (Additional file 2: Movie S1) and did mounting (attempted or successful) within the first 3 min of exposure. Consistent with a previous report [20], the lack of sexual behaviors was clearly apparent in *Cnga2*-null male mice (Additional file 2: Movie S1). Most of the *Cnga2*-mutant mice (7 out of 9 mice) did not initiate the exploration of female anogenital or facial regions. Instead, mutant male mice exhibited only occasional sniff-like behaviors often resembling grooming behaviors. We did not observe any mounting behavior in the *Cnga2*- null male mice during presentation of female mice for 30 min (data not shown). Nonetheless, sniffing/sniff-like behavior was observed within the first 3 min of exposure both in wild type mice and *Cnga2*-null mice (Additional file 2: Movie S1). Neuronal activation in the OB was strikingly lower in those mutant mice (Figure 5B1’, B2’) compared to wild type male littermates (Figure 5B1, B2). Interestingly, a few (2 out of 9) *Cnga2*-null male mice showed positive sexual behaviors which were practically indistinguishable from the wild type behaviors. Those mutants started chemoinvestigation (sniffing/licking) of the female anogenital areas almost instantly and showed mounting behaviors even within the first minute of exposure (Additional file 3: Movie S2). Despite the arousal of sexual behaviors the strong glomerular activation observed in the OB of wild type mice (arrowheads, Figure 5B1) was absent in those mutants. Nevertheless, *c-fos* and other IEGs were induced in a small fraction of OB granule cells in the mutant mice (arrows, Figure 5B1”, B2”); albeit the induction was noticeably lower than that in wild type mice (arrows, Figure 5B1, B2).

Conspecific odor cues considerably induced expression of IEGs in the mouse AOB (arrows, Figure 5B3, B4, compare Figure 5A2). Even in the *Cnga2*-null male mice, a substantial IEG induction was observed in the AOB after exposure to the female stimuli (arrows, Figure 5B3”, B4”). Consistently, IEG induction was lower in the mutants which did not show any apparent sexual behavior (arrows, Figure 5B3-B4”).

We further analyzed neuronal activation in other brain regions of the *Cnga2*-null male mice which were exposed to female mice (Figure 5B5-B8”). We found that induction of *c-fos* expression was very low in the PC of the *Cnga2*-null mice which did not show sexual behaviors (arrows, Figure 5B5’, B6”). In rodents, exposure to estrous odors increased Fos immunoreactivity in the medial amygdala [39] and the medial amygdala was found to regulate attraction to female odor cues [40]. We found that both in wild type mice and *Cnga2* mutants which interacted with female mice, a conspicuous induction of IEGs occurred in the posterodorsal part of the medial amygdaloid nucleus (MePD) (arrows, Figure 5B7, B8, B7”, B8”). Expectedly, the IEG induction in the MePD was much lower in the *Cnga2*-null male mice which did not show sexual behaviors (arrows, Figure 5B7’, B8’). These results provide the evidence that tracing IEG induction by ISH can detect differences in brain activities, with high spatial sensitivity, which correspond to individual behavioral differences. These results also indicate that the lack of amygdaloid activation following presentation of the female sexual stimuli may contribute to the diminished sexual behaviors observed in the majority of *Cnga2*-null male mice.

### TMT exposure activates the OB in *Cnga2*-null mice without eliciting avoidance

**Figure 6:**
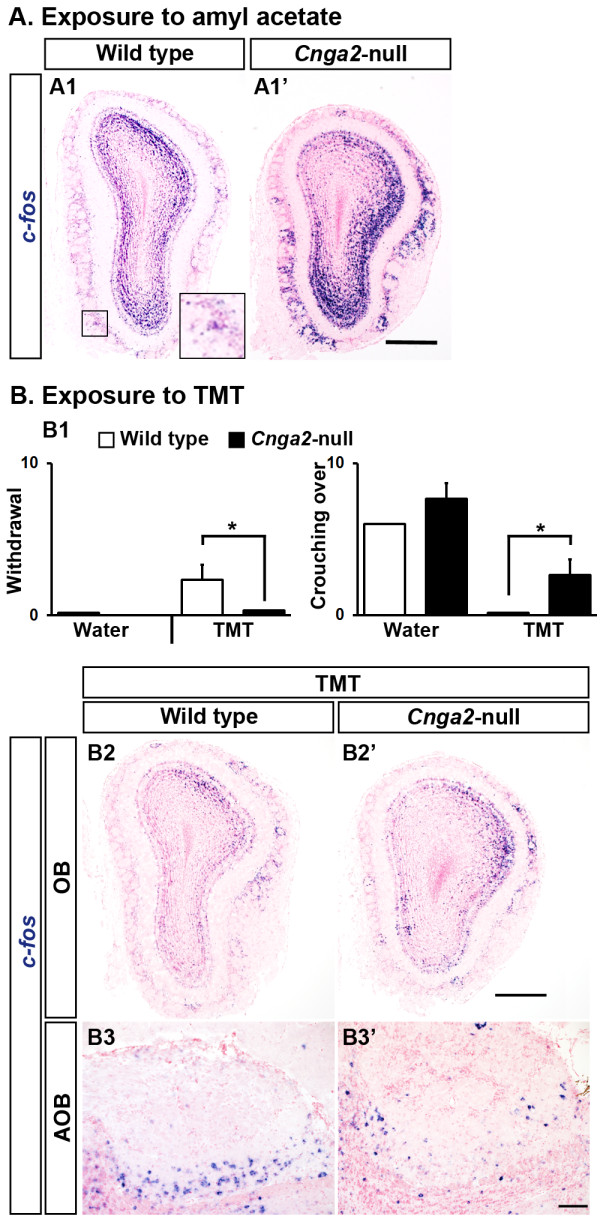
**Neuronal activation in response to amyl acetate and TMT in *Cnga2*-null mice. A.** A neutral odorant, amyl acetate, robustly induced *c-fos* expression in the OB in both wild type (A1) and *Cnga2*-null (A1’) mice. Inset in A1 shows magnified view of the boxed area. **B**. Responses of mice after presentation of TMT, a predator odor from fox. TMT-induced avoidance behaviors were present in wild type mice but absent in *Cnga2*-null mice (number of withdrawal, B1 left). Unlike wild type mice, *Cnga2*-null mice showed increased investigating behaviors for TMT (number of crouching over, B1 right). After exposure to TMT for 30 min, expression of *c-fos* was induced in both wild type and *Cnga2*-null mice in the OB (B2, B2’, respectively) and the AOB (B3, B3’, respectively). Scale bars: (A1, A1’, B2, B2’) 500 μm, (B3-B3’) 100 μm.

We then sought to know if a strong odorant, amyl acetate, can trigger glomerular activation in the *Cnga2*-null OB in our experimental conditions. To our surprise, we observed robust activation of a large number of glomeruli by the emergence of dense *c-fos* mRNA signals in periglomerular cells and in the mitral/tufted cell layer and the granule cell layer below the activated glomeruli (Figure 6A). Interestingly, *c-fos* induction was stronger in the mutant OB, predominantly in the ventrolateral aspects, compared to that in the wild type OB (Figure 6A).

We next exposed the *Cnga2*-null mice and wild type littermates to the predator odor TMT which produces avoidance behaviors in rodents [41]. For behavioral analyses we introduced a piece of filter paper soaked with distilled water or TMT in the mouse cage and observed avoidance behaviors such as stretch attend posture (the animal approaches and sniffs the filter paper with flat back and stretch neck) and withdrawal (the mouse approaches without contact and immediately withdraws from the stimulus) and non-avoidance behaviors such as crouching over object and catching (the mouse takes the filter paper in its mouth) [42]. TMT-induced avoidance behaviors, as quantified by the events of withdrawal, were present in wild type mice but practically absent in *Cnga2*-null mice (Figure 6B1, Additional file 4: Movie S3). In contrast, *Cnga2*-null mice showed non-avoidance behaviors including increased investigation and crouching over the TMT-soaked filter paper unlike their wild type littermates (Figure 6B1, Additional file 4: Movie S3). We then checked IEG expression levels in the mice which were exposed to TMT for 30 min. TMT strongly induced *c-fos* mRNA expression in the wild type OB (Figure 6B2). Notably, we also observed strong induction of IEGs in the OB and the AOB of *Cnga2*-null mice (Figure 6B2’, B3’, respectively) despite the absence of predator odor-induced avoidance response (Figure 6B1). Taken together, our results suggest that the predator odor TMT can strongly activate the main olfactory system in *Cnga2*-null mice although such activation seemed to fail to produce typical avoidance response.

## Discussion

### Detection of neuronal activity using ISH of IEGs

Tracing IEG expression has been proved to be a very reliable and powerful tool for visualization of neuronal activities. In this study we compared mRNA expression patterns of ten IEGs using the ISH method. We found that these IEGs, which included both the transcription factors and effectors, were expressed at low levels in different brain regions in mice at the basal condition (Figure 1). We observed differential expression patterns of these activity-dependent genes in different cell layers of the mouse OB. Interestingly, all these genes were induced significantly in the OB after exposure of the mouse to a given odorant, presumably due to stimulation of the olfactory sensory pathway. However, an increasing number of studies indicate that centrifugal innervation can substantially modulate odor processing in the OB [11-14]. We observed IEG induction in spatially restricted regions in response to propionic acid whereas amyl acetate triggered global induction in the OB (Figures 1, 2 and 3). Therefore, we cannot rule out the possibility that central inputs had role in activation of a large number of OB granule cells we observed in some cases, for instance, after amyl acetate exposure.

The basic helix-loop-helix (bHLH)-PAS transcription factor Npas4 has been previously identified as a critical factor in regulation of inhibitory synapse development on excitatory neurons [43] and recent reports indicate that the *Npas4* gene is involved in learning and memory [44,45]. Expression of *Npas4* mRNA was found to be increased by membrane depolarization in vitro and by 1 h light stimulation in vivo in the visual cortex of dark-reared mice [43]. Our in vivo results indicate that the basal expression of *Npas4* is very low in the mouse OB and a brief olfactory stimulation is sufficient to induce this gene rapidly and transiently in the mouse brain (Figure 1I1-I2’, Figure 2).

We found that the induction of both *c-fos* and *Egr1* took place in a greater number of cells in the OB compared to that of other IEGs, although the basal expression of *Egr1* was slightly higher (Figure 1A1-A2’, D1-D2’). The rapid induction and the wider coverage of *c-fos* expression in different subtypes of cells explain the versatile use of *c-fos* in IEG mapping. Nevertheless, a different IEG may be suitable in a particular experimental setup depending on the neuronal cell type or the stimuli under consideration. For instance, in a recent study Isogai *et al.* (2011) compared the expression of several IEGs in mouse vomeronasal organ and found that the *Egr1*, but not the *c-fos*, was induced robustly following sensory stimulation [46]. Likewise, our results suggest that *Npas4* would be a suitable marker for identifying activation of granule cells in the mouse OB.

### Sexual behaviors in *Cnga2*-null male mice

Previous studies indicated that inactivation of the main olfactory system considerably affects sexual behaviors in male mice [34]. This view has been substantiated by the observation of significant deficits in sexual behavior in male *Cnga2*-null mice [20] since CNGA2 is essential for signal transduction in most of the MOE neurons [15,47,48]. Consistently, we observed that in *Cnga2*-null male mice female sexual stimuli failed to activate the OB and did not initiate sexual behaviors although the IEGs were significantly induced in the AOB. However, there are individual differences and in a longer mating assay Mandiyan *et al.* (2005) found that a female mouse cohabitating with mutants was plugged once and gave birth [20]. We also observed significant sexual arousal in a few *Cnga2*-mutant male mice (see results). This raised the possibility that a CNGA2-independent signaling pathway(s) can activate the OB to initiate sexual behaviors. Our study supports this idea, although it contradicts with the suggestion made by Mandiyan *et al.* (2005) that the sub-population of MOE neurons which use alternative signaling pathway cannot initiate mating responses [20]. We observed induction of IEGs in a significant number of OB granule cells and mitral/tufted cells in the *Cnga2*-null mice which initiated mating behaviors when exposed to estrous female mice (Figure 5B1”, B2”). Previously it has been suggested that the transient receptor potential channel M5 (TRPM5)-expressing OSNs which project to the ventral OB are involved in pheromone signaling in CNGA2-defective mice [49]. In the *Cnga2* mutants we observed stronger induction of IEGs in the dorsal OB (arrows, Figure 5B1”, B2”) in addition to the weaker ventral induction. Therefore, another set of OSNs targeting glomeruli in the dorsal OB may participate in transmitting olfactory signals sufficient to initiate mating behaviors in CNGA2-deficient mice. However, we cannot rule out the possibility that the sexual arousal observed in a few *Cnga2*-null male mice might have been initially triggered by sensory modalities other than olfaction, for instance, visual and/or auditory stimuli, which activated centrifugal inputs to the OB and induced IEGs predominantly in the granule cell layer (Figure 5B1”,B2”) secondary to the activation of the accessory olfactory system (Figure 5B3”,B4”).

### Strong glomerular activation in the OB of anosmic *Cnga2*-null mice

Using ISH we compared the expression level of IEGs in the olfactory system of *Cnga2*-null mice and wild type control mice. We found that the environmental olfactory stimuli in usual laboratory conditions produce significant neuronal activities in the OB of wild type mice whereas the IEG expression levels were remarkably lower in the OB of *Cnga2*-null mice (Figure 5A).

Previously Lin *et al.* (2004) found that CNGA2-deficient mice detected some odorants and the authors suggested cAMP-independent pathways for the observed responses [21]. Later, Munger and colleagues (2007) demonstrated that GC-D neurons, which lack CNGA2 and several other components of the canonical odor transduction pathway and axons of which innervate the necklace glomeruli, can utilize a cGMP-dependent signaling cascade for chemosensory transduction [38]. In those previous studies only a small subset of glomeruli including the necklace glomeruli were found to be activated by the suggested CNGA2-independent signaling pathway(s) [21,38]. In contrast, we observed that amyl acetate robustly induced *c-fos* mRNA expression in the OB of *Cnga2*-null mice, notably at the ventrolateral OB, in a large number of glomeruli which could include, but apparently not limited to, the necklace glomeruli (Figure 6A1’). We also observed that TMT, a predator odor from fox which produces fear responses in wild type mice, induced the expression of IEGs very strongly in the OB (Figure 6B2’) without eliciting any obvious fear response in *Cnga2*-null mice. Previously Kobayakawa *et al.* (2007) found that the mice in which the OSNs were ablated specifically in the dorsal olfactory epithelium lacked innate fear response to TMT even though the mice could detect the odorant [30]. They proposed the existence of hard-wired circuits in the mammalian olfactory system for processing innate responses [30,50]. Our results indicate that a CNGA2-dependent signaling pathway may be essential for the mouse olfactory circuits to initiate innate fear responses.

In our experiments odorant concentrations were high since pure liquid odorants were introduced in the mouse cage. Previously it has been suggested that a cAMP-independent pathway(s) contributed in the EOG responses observed in the MOE of *Cnga2*-null mice exposed to odorants at relatively higher concentrations [21]. Olfactory neurons expressing TRPM5 can detect the chemicals involved in animal communication and TRPM5-expressing OSNs project mainly to the ventral OB [49]. Indeed, we observed strong *c-fos* induction in a large number of glomeruli mainly in the ventral OB in *Cnga2*-null mice exposed to amyl acetate (Figure 6A1’). In addition, a predator odor TMT strongly activated spatially segregated glomeruli in mutants (Figure 6B2’). However, in addition to direct peripheral inputs via OSNs, there could be other possibilities which might contribute to the odor-induced glomerular activation observed in *Cnga2*-null mice. Centrifugal inputs are known to modulate neuronal activities in the rodent OB, predominantly in the granule cell layer [11-14] and might have contributed in the odorant-induced IEG inductions observed in the present study. Although *Cnga2*-null mice appeared normal in several behavioral tests including grooming [20,51], the size of the OB is apparently smaller in the mutants and alteration in brain development has been suggested [37]. Schaefer *et al.* (2002) reported collateral innervation of the olfactory epithelium and OB by some trigeminal ganglion cells in rats [52,53]. Trigeminal activation was found to inhibit olfactory responses [52,54] and thus, the role of trigeminal activation might be insignificant for IEG induction in our experiments. It was interesting that amyl acetate-induced *c-fos* expression was stronger in CNGA2-deficient mice compared to wild type mice and may suggest impaired peripheral adaptation in glomeruli [55] and/or reduced presynaptic inhibition of OSNs [56] in mutants although further studies will be needed to decode the observed phenomena. However, without the CNGA2 subunit there is no functional CNG channel for transduction of olfactory signals in most of the MOE neurons [47,48]. Together, our data provide support for the idea that in addition to the CNGA2-dependent pathway other alternative signaling pathways participate in signal transduction in the mouse main olfactory system.

## Conclusions

In this study we performed ISH analysis to confirm odor-evoked induction of a number of IEGs and show for the first time that in vivo olfactory stimulation can strongly induce the neuronal IEG *Npas4* in the mouse OB. We provide evidence that some odorants can produce strong glomerular activation in the *Cnga2*-null mice in which the olfactory cAMP signaling pathway is almost completely perturbed suggesting involvement of CNGA2-independent signaling pathway(s) for processing olfactory information. Furthermore, our findings advocate that the ISH probe set we established for IEG tracing can be very useful to visualize neuronal activity with high spatial resolution.

## Methods

### Mice

Pregnant ICR mice were purchased from Japan SLC, Inc. *Cnga2* mutant mice (JAX Mice stock number 002905) originated from Dr. John Ngai lab [19] were kindly provided by Dr. Hitoshi Sakano [57]. Since the *Cnga2* gene localizes on X chromosome, *Cnga2*-null male mice were obtained by crossing wild type male mice and heterozygous female mice. Although most of the *Cnga2*-null mice die during early postnatal period, a few rare survivors can grow until adulthood. At least three wild type mice and two *Cnga2*-null mice were analyzed for each condition. For *Cnga2*-null mice, wild type male littermates were used as controls. All measures were taken to minimize pain or discomfort to the mice. All animal procedures were carried out following the guidelines of Kumamoto University and Niigata University.

### Odorant exposure

Mice were exposed to overhead airflow for 2 h in a clean cage without food and water before the odorant exposure. Undiluted odorants were poured into a 1.5-ml microcentrifuge tube attached to the wall of the mouse cage. Following odorants were used: Amyl acetate/Pentyl acetate (60 μl, Wako, Japan), Propionic acid (60 μl, Sigma-Aldrich) and 2, 3, 5-trimethyl-3-thiazoline (TMT) (30 μl, Contech, Canada). If not mentioned otherwise, mice were exposed to the test odorant continuously for 30 min and sacrificed immediately. For analyzing IEG induction a mouse was tested only once to minimize effects of learning.

### Mating assay

Mice were habituated in the test cage for 2 h as described above. A wild type estrous female mouse was presented for 30 min to an individual test mouse with no prior sexual experience. Sexual behaviors (sniffing, mounting and intromission) of the male mouse were observed and the test mouse was sacrificed at the end of the 30-min exposure. Sexual behaviors were considered to be present if the test mouse did mounting (attempted/successful) at least once during the 30-min period.

### TMT-induced avoidance test

Mice were habituated for approximately 10 min in the test cage (30.5 × 20 × 13 cm, without food, water, and lid) followed by the 3-min test period. Then a piece of filter paper (~2 cm × 2 cm) soaked with distilled water (control) or TMT was introduced at one end of the cage. Avoidance and non-avoidance behaviors were observed [42]. Avoidance was counted as the number of events of withdrawal (the mouse approaches the odorant without contacting it, immediately withdrawing from it) and non-avoidance behavior was counted as the number of events of crouching over (the mouse investigates and crouches over the filter paper) [42]. Three *Cnga2*-null male and 3 wild type littermate male mice were used for this test. The test was done twice with intervals of at least 3 days.

### In situ hybridization (ISH)

Sections were prepared from frozen tissue blocks and ISH was performed as described [58]. Briefly, 20-μm coronal tissue sections were digested with Proteinase K (1 μg/ml) for 75 min and post-fixed in 4% PFA. After pre-hybridization, specimens were incubated overnight at 65°C with digoxigenin (DIG)-labeled riboprobes (Information on ISH probes is provided in Additional file 5: Table S1). Following washes, blocking was done by 1% sheep serum, 1% bovine serum albumin (BSA) and 0.1% Triton X-100 in phosphate-buffered saline (PBS). Afterwards, samples were incubated overnight at 4°C with alkaline phosphatase-conjugated anti-DIG antibody (1:2000, Roche Diagnostics, Germany). Sections were washed in MABT (100 mM Maleic acid, 150 mM NaCl, 0.1% Tween 20) and then in alkaline phosphatase buffer (100 mM NaCl, 100 mM Tris–HCl, pH 9.5, 50 mM MgCl_2_, 0.1% Tween 20, 5 mM Levamisole). Tissue sections were treated with NBT/BCIP (Roche) mixture at room temperature in dark for color development. After ISH staining, sections were counterstained by nuclear fast red.

### Quantification of IEG expression levels

Images of stained coronal sections of the olfactory bulb were captured with an Olympus microscope and digital camera system (BX53 and DP72; Olympus, Tokyo, Japan). Quantification was performed using Adobe Photoshop CS5 Extended (version 12.0.4 × 64, Adobe Systems Incorporated) adapting the techniques described previously [59,60]. The glomerular layer, the mitral cell layer and the granule cell layer were selected separately by the Lasso tool. Total number of pixels and the number of pixels positive for ISH signals were counted using the Histogram tool. Signal intensity (arbitrary unit) of IEGs was calculated as the percentage of area positive for ISH signals in respective layers of the olfactory bulb. Data were plotted in column charts where columns represented mean ± SEM. Seven to eight bulbs (approximately from + 4.5 mm bregma to + 4 mm bregma) from two to three mice were analyzed.

### Statistical analysis

Student's *t*-test was performed to compare means. Difference between groups was considered highly significant (**) when p≤ 0.01 and significant (*) when p≤ 0.05.

## Abbreviations

ACIII: Adenylyl cyclase type III; AOB: Accessory olfactory bulb; AON: Anterior olfactory nucleus; Cnga2: Cyclic nucleotide-gated channel subunit A2; EOG: Electro-olfactogram; G_olf_: Olfaction-specific G protein; IEG: Immediate early gene; ISH: In situ hybridization; MePD: Dorsomedial part of the medial amygdaloid nucleus; MOE: Main olfactory epithelium; OB: Olfactory bulb; OR: Olfactory receptor; OSN: Olfactory sensory neuron; PC: Piriform cortex; TMT: 2, 3, 5-trimethyl-3-thiazoline.

## Competing interests

The authors declare that they have no competing interests.

## Authors' contributions

AKB carried out the experiments, performed statistical analysis and drafted the manuscript. KW participated in the design of the study, and helped to analyze the data and to draft the manuscript. MY participated in the design of the study and revised the manuscript critically. NT participated in the design of the study and revised the manuscript. HT conceived of the study, and participated in its design and coordination and helped to draft the manuscript. All authors read and approved the final manuscript.

## Supplementary Material

Additional file 1 **Figure S1. **Strong residual activity at the necklace glomeruli in *Cnga2*-null mice. Figure shows horizontal sections of the OB. Expression of *Th*, a marker of afferent activity, was significantly reduced in most of the OB glomeruli in *Cnga2*-null mice (B1) compared to that of wild type mice (A). However, strong *Th* expression was observed in a small number of glomeruli (B1, inset), presumably the necklace glomeruli which express *Pde2* (B2, inset). In *Cnga2*-null mice *c-*fos expression was almost absent in the OB. However, strong *c-fos* signals appeared in a few glomeruli (B3, inset). Scale bar: 200 μm. Click here for file

Additional file 2 **Movie S1.** Behavioral responses of wild type and Cnga2-null male mice after presentation of estrous female mice. Click here for file

Additional file 3 **Movie S2.** Positive sexual behavior in *Cnga2*-null male mice. Click here for file

Additional file 4 **Movie S3.** TMT-induced avoidance test. Click here for file

Additional file 5 **Table S1.** Information on ISH probes. Click here for file
