# Supplementary figures and images for: Visualization of odor-induced neuronal activity by immediate early gene expression
Source: BMC Neurosci. 2012 Nov 5;13:140. doi: 10.1186/1471-2202-13-140 (PMC3538715; doi:10.1186/1471-2202-13-140)

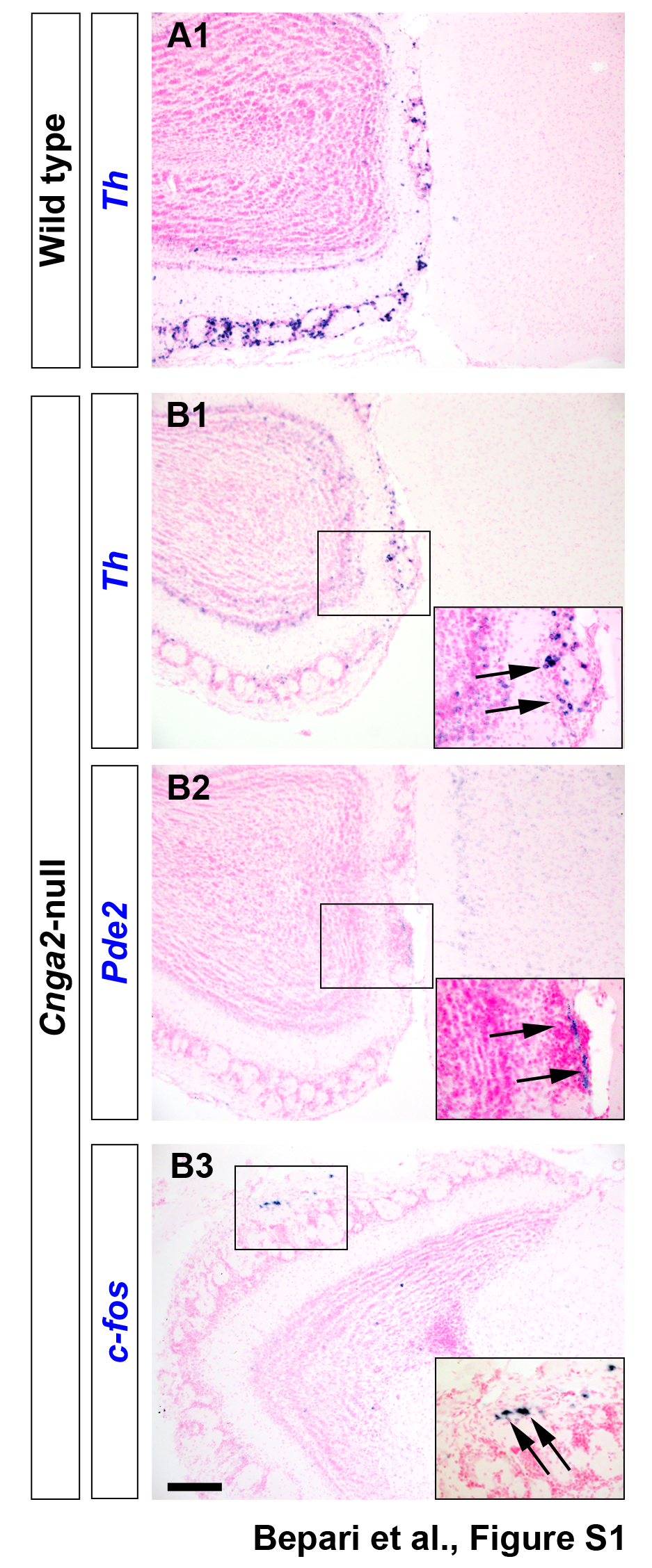

Supplement: Additional file 1 — Figure S1. Strong residual activity at the necklace glomeruli in Cnga2-null mice. Figure shows horizontal sections of the OB. Expression of Th, a marker of afferent activity, was significantly reduced in most of the OB glomeruli in Cnga2-null mice (B1) compared to that of wild type mice (A). However, strong Th expression was observed in a small number of glomeruli (B1, inset), presumably the necklace glomeruli which express Pde2 (B2, inset). In Cnga2-null mice c-fos expression was almost absent in the OB. However, strong c-fos signals appeared in a few glomeruli (B3, inset). Scale bar: 200 μm. [file 1471-2202-13-140-S1.tiff]
