# Supplementary material for: Visualization of odor-induced neuronal activity by immediate early gene expression
Source: BMC Neurosci. 2012 Nov 5;13:140. doi: 10.1186/1471-2202-13-140 (PMC3538715; doi:10.1186/1471-2202-13-140)
Supplement: Additional file 5 — Table S1. Information on ISH probes. [file 1471-2202-13-140-S5.doc]

**Additional file 5**

**Table S1: Information on ISH probes**

| **Gene symbol** | **Remarks**  **(Genbank accession number)** | **Gene Name**  **Synonyms** | **Forward primer**  **Reverse primer** | **Vector** | **Restriction enzyme for AS probe** | **RNA polymerase for AS probe** |
| --- | --- | --- | --- | --- | --- | --- |
| ***Arc*** | (AF162777) | **activity regulated cytoskeletal-associated protein**  Arc3.1 | 5’-CGCAGCACCGACGACCAGAT-3’  5’-GACCAGGGCAGACAGATGAG-3’ | p123T | BamHI | T7 |
| ***c-fos*** | EST clone  (BC029814) | **FBJ osteosarcoma oncogene**  Fos, cFos | None | pCMV-SPORT6 | EcoRI | T7 |
| ***c-jun*** | EST clone  (BC094032) | **Jun oncogene**  Jun, Junc | None | pCMV-SPORT6 | EcoRV | T7 |
| ***Egr1*** | EST clone  (NM_007913) | **early growth response 1**  Egr-1, Krox-1, Krox-24, Zif268 | None | pCMV-SPORT6 | EcoRI | T7 |
| ***Egr3*** | (NM_018781) | **early growth response 3**  Pilot | 5’-CAATCTGTACCCCGAGGAGA-3’  5’-CCACAGAACTCACAGGCAAA-3’ | p123T | BamHI | T7 |
| ***Fosb*** | (NM_008036) | **FBJ osteosarcoma** oncogene B | 5’-CGGTCTCGGGGAAATGCCCG-3’  5’-GCGAGTTCAGCGGGTCGGAC-3’ | pCR II | EcoRV | Sp6 |
| ***Jun-B*** | EST clone  (BC003790) | **Jun-B oncogene** | None | pCMV-SPORT6 | EcoRV | T7 |
| ***Nor1*** | A kind gift from Dr Levesque, Université de Montréal  Ref. J Pharmacol Exp Ther 313: 460-473, 2005. (NM_015743) | **nuclear receptor subfamily 4, group A, member 3**  Nr4a3, NOR-1 | See ref. | pBluescript SK+ | HindIII | T3 |
| ***Npas4*** | (NM_153553) | **neuronal PAS domain protein 4**  Nxf | 5’-TCTCACTGTGCGCCAGCAGC-3’  5’-CCACGCCCTGAGCCAACTGG-3’ | pCR II | BamHI | T7 |
| ***Nr4a1*** | (NM_010444) | **nuclear receptor subfamily 4, group A, member 1**  NGFI-B, Nur77 | 5’-GCGGAACCGCTGCCAGTTCT-3’  5’-GTTGGAGGCTCGCCCAGCTG-3’ | pCR II | BamHI | T7 |
| ***Pde2***  **(*Pde2a*)** | A kind gift from Dr. Joseph Beavo,  University of Washington  Ref. PNAS 94: 3388-3395, 1997  (NM_001008548) | **phosphodiesterase 2A, cGMP-stimulated**  CGS-PDE; cGSPDE | See ref. | pcDNA3 | HindIII | Sp6 |
| ***Th*** | (NM_009377.1) | **tyrosine hydroxylase** | 5’-TGTCACGTCCCCAAGGTTCA-3’  5’-CCAAGAGCAGCCCATCAAAG-3’ | p123T | BamHI | T7 |
